# Acylcarnitines Are Associated with Metabolic Syndrome and Hypertension in Two Contrasted Obese Populations

**DOI:** 10.3390/nu18071137

**Published:** 2026-04-01

**Authors:** Nicodème Chabi, Claire Nominé-Criqui, Almut Heinken, Didier Quilliot, Laurent Brunaud, Zhen Li, Elise Jeannesson-Thivisol, Ambaliou Sanni, Olivier Ziegler, Jean-Louis Gueant, Rosa-Maria Guéant-Rodriguez

**Affiliations:** 1 Inserm UMR 1256 NGERE (Nutrition-Genetics-Environmental Risks), Université de Lorraine, F-54000 Nancy, France; nicodeme.chabi@gmail.com (N.C.); c.nomine-criqui@chru-nancy.fr (C.N.-C.);; 2 Laboratory of Biochemistry and Molecular Biology, University of Cotonou, Cotonou 04 BP 0320, Benin; 3 Inserm UMR 1256 NGERE (Nutrition-Genetics-Environmental Risks), Multidisciplinary Unit of Metabolic and Endocrine Surgery, Department of Gastrointestinal, Metabolic, and Cancer Surgery (CVMC), CHRU-Nancy, University of Lorraine, F-54000 Nancy, France; l.brunaud@chru-nancy.fr; 4 Inserm UMR 1256 NGERE (Nutrition-Genetics-Environmental Risks), Department of Endocrinology Diabetology and Nutrition, CHRU-Nancy, University of Lorraine, F-54000 Nancy, France; 5 Inserm UMR 1256 NGERE (Nutrition-Genetics-Environmental Risks), Reference Medical Biology Laboratory (LBMR), Department of Biochemistry, Molecular Biology and Nutrition, CHRU-Nancy, University of Lorraine, F-54000 Nancy, France; 6 Joan Klein Jacobs Center for Precision Nutrition and Health, Cornell University, Ithaca, NY 14853, USA; 7 Institute for Obesity Research, Tecnologico de Monterrey, Monterrey 64849, Mexico

**Keywords:** metabolic syndrome, obesity, hypertension, acylcarnitines, β-oxidation

## Abstract

**Background**: Metabolic syndrome (MetS) is a constellation of components that includes type 2 diabetes/hyperglycemia, central obesity, hypertension, and dyslipidemia. Its prevalence is increasing dramatically in Africa, where it is predominant in obese females. Incomplete fatty acid β-oxidation is among the complex mechanisms that increase MetS risk. However, it remains unclear whether MeS components are associated with increased acylcarnitine categories. **Objective**: The aim of this study was to analyze the relationship between acylcarnitines and all components of metabolic syndrome in females with obesity from two populations with distinct ethnicities and dietary habits. **Methods**: We investigated the association of obesity (BMI > 35) with acylcarnitines determined by LC–MS/MS with MetS components in women from Benin, West Africa, and French women. Acylcarnitines and biological and clinical outcomes of MetS according to International Diabetes Federation (IDF) criteria were assessed in 428 ambulatory women recruited at the market of Dantokpa, Cotonou, and 220 women of Aldepi/Obesepi cohort recruited in the North-East of France. **Results**: Compared to those without MetS, we observed an association of short-, medium-, and long-chain acylcarnitines (SC, MC, LC) with MetS (*p* = 0.0001; *p* < 0.0001 and *p* = 0.0004, respectively) in African women and of SC acylcarnitines with MetS (*p* < 0.0001), blood glucose (*p* < 0.001), low HDL-cholesterol (*p* < 0.05) and high triglycerides (*p* < 0.01) in French women. We also observed significant associations of MC and LC total and mono-unsaturated acylcarnitines with hypertension in both African and French populations (*p* < 0.001 and *p* < 0.01, respectively). The independent determinants of systolic blood pressure were age, body mass index, glycemia, long-chain acylcarnitines, LDL-C and HDL-C. **Conclusions**: An association of acylcarnitine indexes of altered SC, MC, LC fatty acid β-oxidation with hypertension was reported in the two contrasted women populations, while an association of altered SC fatty acid β-oxidation with glucose and hypertriglyceridemia was reported in French women only. The association of altered acylcarnitine indexes with high SBP could be related to the effects of impaired β-oxidation on endothelial dysfunction. This study was registered at clinical trials.gov as NCT02663388.

## 1. Introduction

Metabolic syndrome (MetS) is an association of interrelated risk factors of cardiovascular disease (CVD) and type 2 diabetes mellitus (DT2M) that is highly prevalent in people with obesity. The components of MetS include high levels of glucose and triglycerides, high blood pressure, hypertriglyceridemia, low high density lipoprotein cholesterol levels, and obesity. The prevalence of MetS in adults from undeveloped countries ranges between 20 and 27% [1,2,3].

Mitochondrial dysfunction is believed to play a central role in the development of metabolic syndrome, particularly in the onset of insulin resistance [4]. Two complementary hypotheses have been proposed to explain this link. The first suggests that reduced lipid oxidation leads to intracellular lipid accumulation, promoting lipotoxicity and, consequently, insulin resistance [5]. The second emphasizes the concept of “excess fuel,” whereby an oversupply of lipids overwhelms metabolic capacity and contributes to insulin resistance [6]. In both cases, disrupted lipid homeostasis—resulting from an imbalance between lipid availability/storage and lipid use/oxidation—is considered a key mechanism. Blood acylcarnitine concentrations, which serve as markers of β-oxidation, may reflect impairments in this pathway. Previous studies investigating the relationship between β-oxidation markers and obesity have primarily compared lean and obese individuals [7,8], and some have also explored the impact of a Western diet on metabolic profiles [9,10]. In patients with metabolic syndrome (MetS), overnutrition is associated with elevated circulating non-esterified fatty acids (NEFA) and increased skeletal muscle uptake of NEFA.

The profile of acylcarnitines is classically used for the diagnosis of inherited metabolic diseases. Abnormal acylcarnitine concentrations and indexes are indicative of impaired activities of mitochondrial acyl-CoA dehydrogenases in patients with myocardium dysfunction, including very-long-chain acyl-CoA dehydrogenase (VLCAD) and medium-chain acyl-CoA dehydrogenase (MCAD) and the oxidation pathway of short-chain acyl-CoA [11,12]. Little is known about acylcarnitines in obese subjects with metabolic syndrome, including in Afro-American, Afro-African and European populations. Associations have been reported between impaired long-chain fatty acid beta-oxidation and type 2 diabetes mellitus (T2DM) in African-American women and between carnitine and systolic blood pressure in ambulatory Afro-African and Afro-Caucasian men [13,14,15]. Several studies assessed the frequency and clinical and biological determinants of MetS with diseases according to environment and ethnic differences in Sub-Saharan Africa [16,17,18,19]. In Africa, the population is undergoing a nutritional transition due to economical emergency and new habits of nutrition adopted by Western alimentation. France is one of the Western Europe countries with the lowest prevalence of obesity with MetS outcomes [20]. However, no study evaluated and compared the association of the whole spectrum of acylcarnitines with all components of metabolic syndrome in females with obesity from populations with distinct ethnicities and dietary habits.

We, therefore, aimed to investigate whether defective beta-oxidation of fatty acids—as reflected by acylcarnitine’s indexes—is associated with components of metabolic syndrome among contrasted populations despite distinct ethnicities, dietary habits and type of obesity. To address this issue, we analyzed acylcarnitines in 648 women from the Dantokpa cohort of Benin and Aldepi/Obesepi cohort of North-East of France.

## 2. Materials and Methods

### 2.1. Population Samples and Data Collection

The African population study was performed on 35–50-year-old ambulatory women with a very high prevalence of obesity, with BMI > 35, from the Dantokpa central market of Cotonou (Benin, West Africa). Four hundred and twenty-eight (428) women were enrolled. Pregnant women and those who are less than three months postpartum were excluded. The French population consisted of 220 consecutive 35–50-year-old women recruited before bariatric surgery in a multidisciplinary unit for obesity surgery between January 2012 and December 2014 [21] (Supplemental Figure S1b). We consider the women who attended the center at baseline, not those who were selected for bariatric surgery at the end of the assessment. This study was registered at the French Ministry of Health as NCT02663388. The two populations were age-matched (Table 1). In both populations, we limited the exclusion criteria only to pregnancy and post-pregnancy.

All participants provided informed consent to engage in this study. Each local ethics committee approved both cohort studies and conformed with the Declaration of Helsinki.

The recruited women were subjected to a questionnaire providing information on family history, school status, physical activities, height, weight, waist circumference (WC), hip circumference, hypertension, diabetes mellitus, dyslipidemia, and non-cardiovascular diseases. Weights and heights were used to define body mass index (BMI) as a measure of overall or general obesity (BMI > 30 kg/m^2^) according to the World Health Organization. WC was measured using a flexible non-stretch tape to the nearest 0.1 cm at midpoint between the lower rib and the iliac crest while subjects were standing and breathing normally. A validated food questionnaire could not be performed in the DANTOKPA cohort, avoiding any careful comparison of dietary profiling between both populations.

### 2.2. Criteria for Metabolic Syndrome

The International Diabetes Federation (IDF), an umbrella organization of over 230 national diabetes associations in 170 countries, identified five components of metabolic syndrome that related to CVD (abdominal obesity, increased triglyceride concentration, reduced HDL-cholesterol concentration, high blood pressure, and increased fasting glucose concentration) [22]. In agreement with the IDF definition, the subjects were stratified for MetS according to central obesity (defined as waist circumference with ethnicity specific values) and any two of the other four factors.

### 2.3. Sample Size

For sample size calculation, we assumed that the frequency of MetS ranked from 22.3 to 31.5% in Europe and from 15.4 to 28.5% according to different MetS definitions. These assumptions were made according to a recent meta-analysis [23]. We estimated a minimum sample size of 182 subjects for a type I error of *p* < 0.05 and an 80% test power.

### 2.4. Biochemical Analyses

The blood samples were collected at fasting in ice glass and sent to the lab for centrifugation to obtain plasma, serum, and buffy coat. Serum and plasma were obtained by centrifugation at 2.000× *g* for 10 min, frozen, and stored at −20°C until analyses. All materials (plasma, serum, and buffy coat) were sent to Inserm UMRS 1256 N-GERE (Nancy, France) for subsequent analyses. Plasma concentrations of glucose, cholesterol, fasting triglycerides, HDL-cholesterol, CRP (C-reactive protein), albumin, sodium, potassium, chlorine, proteins, urea, creatinine, calcium, phosphorus, bilirubin, iron, ASAT and ALAT were determined by the enzymatic or dry chemistry method using on automata U 2700 Olympus Corporation, Center Valley, PA, USA. LDL-cholesterol values were computed according to the Friedewald formula. The HOMA-IR was calculated using the model proposed by Levy et al. [16]. Vitamin B12, folate, homocysteine and methylmalonic acid (MMA) were determined as described in [12]. Acylcarnitines plasma concentrations were measured by a UPLC Acuity system (Waters SAS, Saint-Quentin-en-Yveline, France) coupled to a mass Spectrophotometer 4000 QTRAP LC–MSMS System (Applied Biosystems MDS SCIEX, Concord, ON, Canada), as described previously [12].

### 2.5. Statistical Analysis

Variables not normally distributed were logarithmically transformed. Categorical variables were first reported in number and percentage, and continuous variables were reported as mean and standard deviation (SD). A chi-square test was used to assess differences in nominal variables. The Mann–Whitney U test was used for continuous variables. Differences in acylcarnitine concentrations between participants with and without metabolic syndrome were further assessed using linear regression models adjusted for age. The associations between indexes based on ratios of acylcarnitine concentrations—indicative of beta-oxidation of SC-, MC-, and LC-acylcarnitines—and continuous variables of MetS components were assessed by Rho-Spearman rank-order correlation analysis. Multiple linear regression analysis was used to assess the independent determinants of acylcarnitines. Multivariate logistic regression analysis considered a model that included age and all the variables that had a *p* value < 0.10. A value of *p* < 0.05 was considered statistically significant. For visualization purposes, circular plot analysis was performed to illustrate associations between acylcarnitines and metabolic syndrome-related variables, using “Sunburst chart”. Correlations were computed using Spearman coefficients, and only statistically significant associations (*p* < 0.05) were retained. Data were analyzed using the STATA 12.1 software (College Station, TX, USA). We performed circos plots for visualization of associations between acylcarnitines in French and African populations, both in the overall respective populations and stratified by MetS status (No MetS and MetS). Circos plots were generated using R software (version 4.5.3, “Reassured Reassurer”, R Foundation for Statistical Computing, Vienna, Austria).

## 3. Results

### 3.1. Clinical and Biological Characteristics of the Two Populations

The African population was composed of 428 females, with a mean age of 41 + 6.2 years; a total of 252 (58.9%) were obese (BMI higher than 30), 85 (19.9%) had a MetS according to IDF definition, nine (2.1%) subjects had a high level of triglycerides, and 221 (51.8%) had low HDL-cholesterol; a total of 152 (35.5%) had high blood pressure (>130 mmHg), and 15 (3.5%) had a high glucose level. The females with MetS were older than those with no MetS (Table 1). We found no significant difference between both groups for fasting glucose, insulin, ASAT and ALAT (Table 1). The median (25–75 quartiles, extreme values) was at 120 mmHg (110–140, 88–222) in the whole population and 118 (110–130, 86–202) and 150 (140–160, 100–220) in subjects without and with MetS, respectively.

The French population was composed of 220 women, with a mean age of 45 + 0.8 years. A total of 134 patients (61.0%) had MetS according to the IDF definition [22], 85 (38.6%) patients had a high level of triglycerides, 164 (74.9%) had low HDL-cholesterol, 85 (38.6%) had hypertension under treatment, and 85 (38.6%) had a high glucose level. The patients with MetS were older than those with no MetS. Compared to African women, they had a significantly higher frequency of treated hypertension and higher levels of fasting glucose, insulin, ASAT and ALAT (Table 1).

### 3.2. Acylcarnitine Concentrations According to Metabolic Syndrome

**Figure 1:**
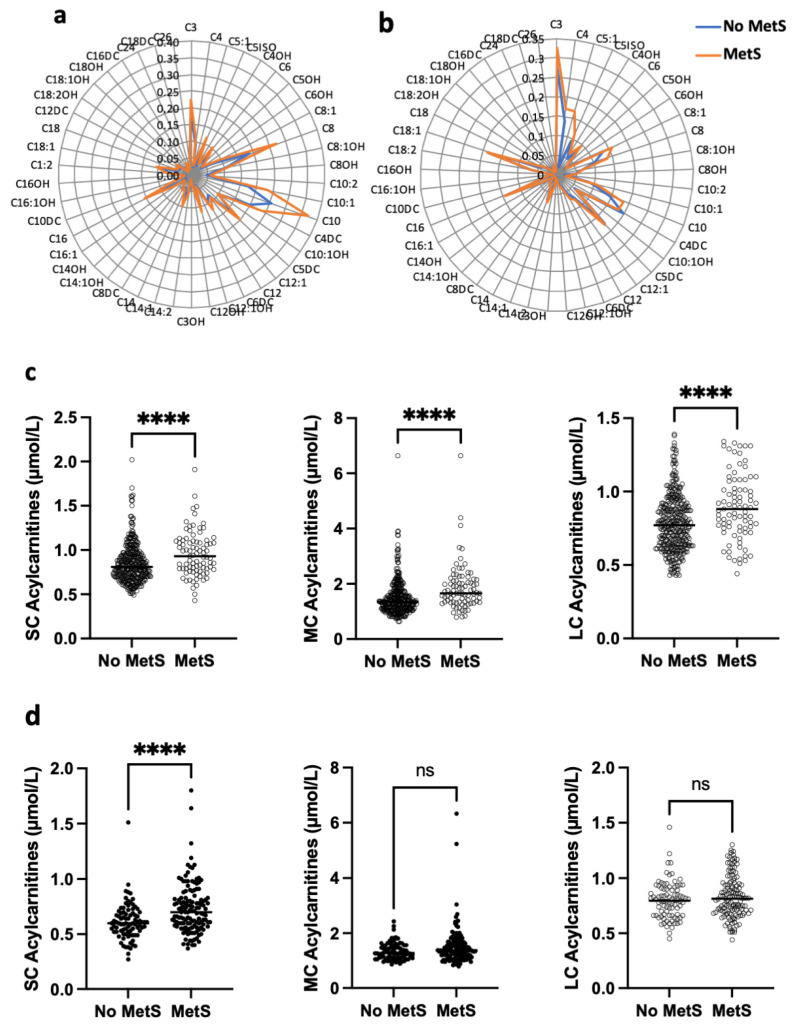
Acylcarnitine concentrations according to metabolic syndrome (MetS). (**a**,**b**) Sunburst chart of acylcarnitine concentrations according to presence or absence of MetS in African (**a**) and French (**b**) populations. (**c**,**d**) Short-, medium- and long-chain acylcarnitines (SC, MC, LC) according to metabolic syndrome (MetS) in African (**c**) and French (**d**) populations. Differences were assessed by Mann–Whitney U test. Statistical significance, ns: non-significant, **** *p* < 0.0001.

The women of the African population with MetS had a higher concentration of total carnitine compared to those without MetS (44.8 ± 10.0 µmol/L vs. 41.4 ± 9.7 µmol/L, *p* = 0.0033); free carnitine (38.2 ± 9.1 µmol/L vs. 35.4 ± 9.1 µmol/L, *p* = 0.0127); and short-, medium- and long-chain carnitines (0.95 ± 0.25 µmol/L vs. 0.85 ± 0.21 µmol/L, *p* < 0.0001; 1.85 ± 0.84 µmol/L vs. 1.48 ± 0.62, *p* < 0.0001; 0.89 ± 0.22 µmol/L vs. 0.78 ± 0.19 µmol/L, *p* < 0.0001, respectively) (Figure 1a,c, Supplementary Table S1).

French women with MetS also had significantly higher concentrations of acylcarnitine categories compared to those without MetS, including total carnitine (47.5 ± 1.0 µmol/L vs. 43.2 ± 1.2 µmol/L, *p* = 0.0037), free carnitine (36.4 ± 0.9 µmol/L vs. 33.0 ± 0.9 µmol/L, *p* = 0.0199), and short-chain acylcarnitines (0.73 ± 0.02 µmol/L vs. 0.61 ± 0.01 µmol/L, *p* < 0.0001). Medium-chain (1.51 ± 0.06 µmol/L vs. 1.35 ± 0.03, *p* = 0.0907) and long-chain acylcarnitines (0.83 ± 0.02 µmol/L vs. 0.80 ± 0.01 µmol/L, *p* = 0.3316) were not statistically different (Figure 1b,d, Supplementary Table S1). Circos plots showed very clear differences of acylcarnitine associations between French and African women but no clear signatures related with metabolic syndrome in any of the two populations (Supplementary Figure S1).

We studied indexes of acylcarnitine concentrations as indicators of flux activities from pathways involved in fatty acid oxidation. We found no change in the activity index of mitochondrial carnitine palmitoyl transferase I in any of populations, using CPT-I ratio C18 + C16)/C0, and no change in the indexes of oxidative degradation of even-numbered fatty acids (C2/C0) ratio) and overall activity of ß-oxidation (C2 + C3)/C0) ratio) (Table 2). In the African women, the higher concentrations of C4, C8 and C14:1 were indicative of decreased activity of short-chain acyl-CoA dehydrogenase (SCAD), MCAD and VLCAD, respectively (Table 2). In contrast, the higher concentration of C4, indicative of decreased activity of SCAD, was the single abnormal index reported in the French population.

### 3.3. Acylcarnitine Concentrations According to Specific Components of Metabolic Syndrome

We investigated the association of SC, MC, and LC acylcarnitines with each MetS component.

**Figure 2:**
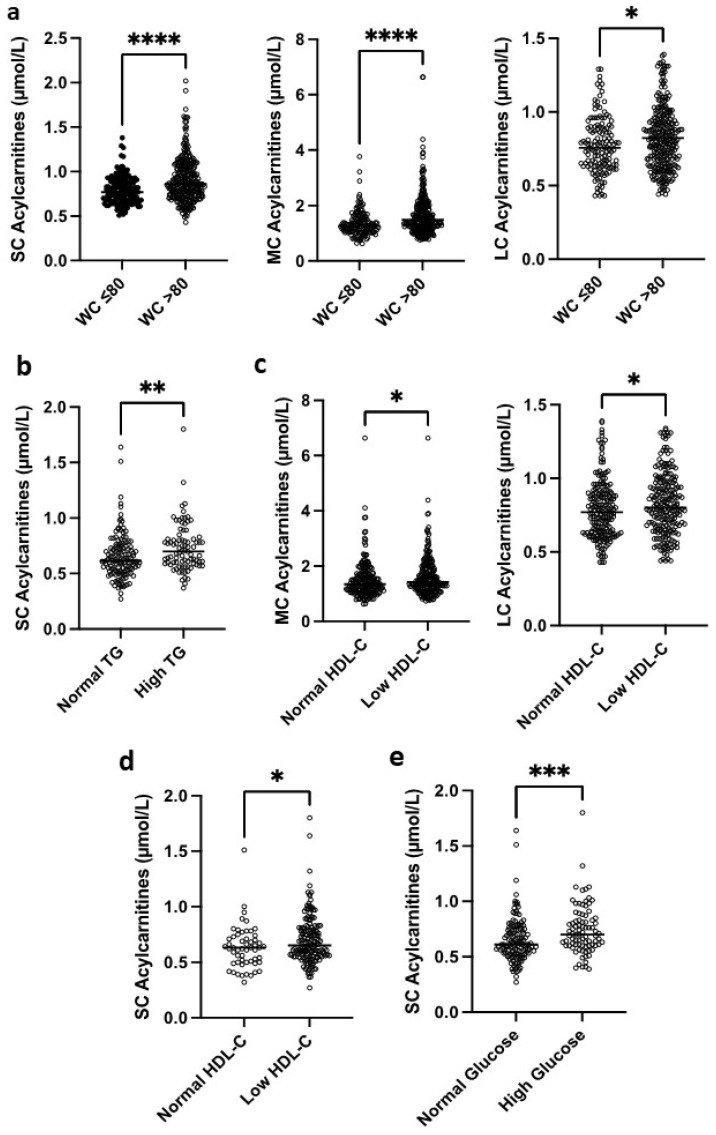
Acylcarnitine concentrations according to parameters related to metabolic syndrome (MetS). (**a**) Short-, medium- and long-chain acylcarnitine (SC, MC, LC) concentrations according to waist-to-hip ratio (WHR > 0.80) in African population. (**b**) SC acylcarnitines according to hypertriglyceridemia (>1.5 g/L) in French population. (**c**) MC, and LC acylcarnitines according to low HDL-cholesterol (HDL-C < 0.5 g/L) in African (**d**) SC acylcarnitines according to low HDL-cholesterol (HDL-C < 0.5 g/L) in French population. (**e**) SC acylcarnitines according to hyperglycemia in the French population. Differences were assessed by Mann–Whitney U test. Statistical significances * *p* < 0.05, ** *p* < 0.01, *** *p* < 0.001, **** *p* < 0.0001.

Central obesity could be evaluated according to the waist-to-hip ratio (WHR > 0.80) in African women but not in French women with morbid obesity. We observed a marked increase in SC and MC acylcarnitines (0.93 ± 0.25 vs. 0.83 ± 0.20 μmol/L, *p* < 0.0001 and 1.70 ± 0.71 vs. 1.47 ± 0.66 μmol/L, *p* < 0.0001, respectively) and a moderate increase in LC acylcarnitines (0.84 ± 0.21 vs. 0.79 ± 0.19 μmol/L, *p* = 0.0375) in African women with a WHR > 0.80 compared with those with a lower WHR (Figure 2a).

Regarding hypertriglyceridemia (>1.5 g/L), we did not observe any significant difference in the level of SC, MC, or LC acylcarnitines in African women, while French women with high triglycerides had a higher level of SC acylcarnitines compared to those with normal triglycerides (0.73 ± 0.21 vs. 0.65 ± 0.20 μmol/L, *p* = 0.0018) (Figure 2b). Among African women, those with low HDL-C levels had significantly higher concentrations of medium-chain (MC) and long-chain (LC) acylcarnitines compared with women with normal HDL-C levels (1.62 ± 0.70 vs. 1.49 ± 0.66 μmol/L, *p* = 0.0178; and 0.83 ± 0.20 vs. 0.79 ± 0.19 μmol/L, *p* = 0.0371, respectively) (Figure 2c). In contrast, in the French population, women with low HDL-C levels showed higher short-chain (SC) acylcarnitine concentrations than those with normal HDL-C levels (0.70 ± 0.21 vs. 0.63 ± 0.19 μmol/L, *p* = 0.0367) (Figure 2d). Significant associations were observed between SC, MC, and LC acylcarnitine levels and high blood glucose or a history of diabetes in African women. However, in French women, those with diabetes or elevated blood glucose levels had significantly higher SC acylcarnitine concentrations compared with non-diabetic women with normal glucose levels (0.74 ± 0.22 vs. 0.64 ± 0.19 μmol/L, *p* = 0.0002) (Figure 2e).

**Figure 3:**
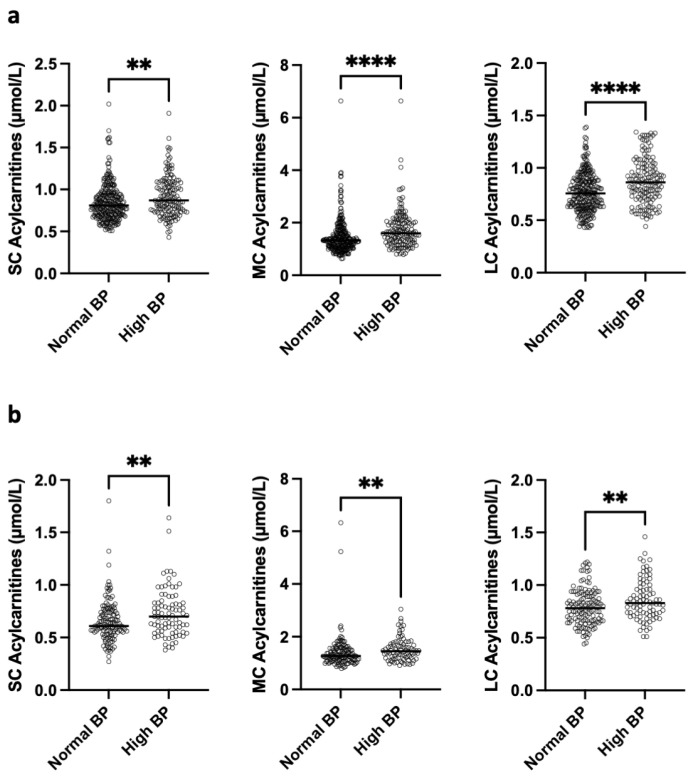
Acylcarnitine concentrations according to systolic blood pressure. (**a**) short chain (SC), medium chain (MC), and long chain (LC) acylcarnitines in African population with normal vs. high systolic blood pressure (BP). (**b**) SC, MC, and LC acylcarnitine concentrations according to normal BP vs. treated hypertension for high systolic blood pressure in French population Differences were assessed by Mann–Whitney U test. Significances ** *p* < 0.01, **** *p* <0.0001.

The most intriguing result was observed in women with hypertension either in French or African women. The hypertension was defined according to any of the following three criteria: diastolic blood pressure ≥ 85 mmHg, systolic blood pressure ≥ 130 mmHg and reported treatment. Among African women, those with hypertension exhibited significantly higher concentrations of short-chain (SC), medium-chain (MC), and long-chain (LC) acylcarnitines compared with normotensive women (0.92 ± 0.23 vs. 0.85 ± 0.21 μmol/L, *p* = 0.0014; 1.74 ± 0.72 vs. 1.46 ± 0.64 μmol/L, *p* < 0.0001; and 0.88 ± 0.21 vs. 0.77 ± 0.18 μmol/L, *p* < 0.0001, respectively) (Figure 3a). A similar pattern was observed in the French population, where hypertensive women also had higher levels of SC, MC, and LC acylcarnitines than those without hypertension (0.73 ± 0.22 vs. 0.66 ± 0.19 μmol/L, *p* = 0.0073; 1.52 ± 0.43 vs. 1.40 ± 0.62 μmol/L, *p* = 0.0026; and 0.86 ± 0.19 vs. 0.79 ± 0.17 μmol/L, *p* = 0.0093, respectively) (Figure 3b). In addition, significant positive correlations were found between SC, MC, and LC acylcarnitine levels and systolic blood pressure (SBP) in African women (*p* < 0.008, *p* < 0.001, and *p* < 0.001, respectively). These correlations could not be assessed in French women because continuous systolic blood pressure (SBP) values were not available. 

We, therefore, examined which individual acylcarnitine species were specifically associated with elevated SBP and/or self-reported hypertension in both populations (Figures S2 and S3). 

**Figure 4:**
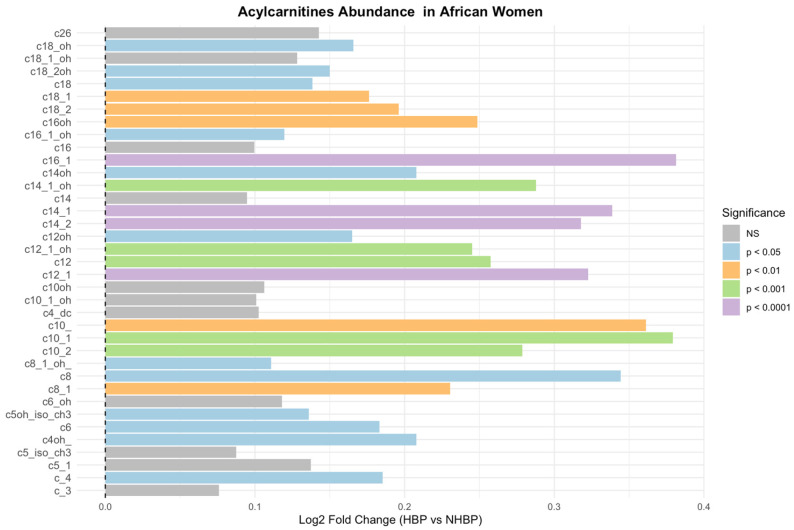
Log2 fold changes in acylcarnitine concentrations between participants with and without high blood pressure are shown. Positive values indicate higher levels in participants with high blood pressure in African population. Colors represent levels of statistical significance based on the Mann–Whitney U test.

**Figure 5:**
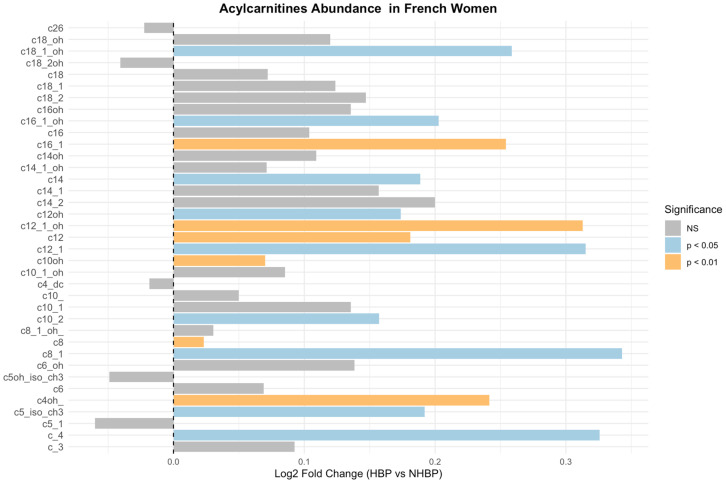
Log2 fold changes in acylcarnitine concentrations between participants with and without high blood pressure are shown. Positive values indicate higher levels in participants with high blood pressure in French population. Colors represent levels of statistical significance based on the Mann–Whitney U test.

**Figure 6:**
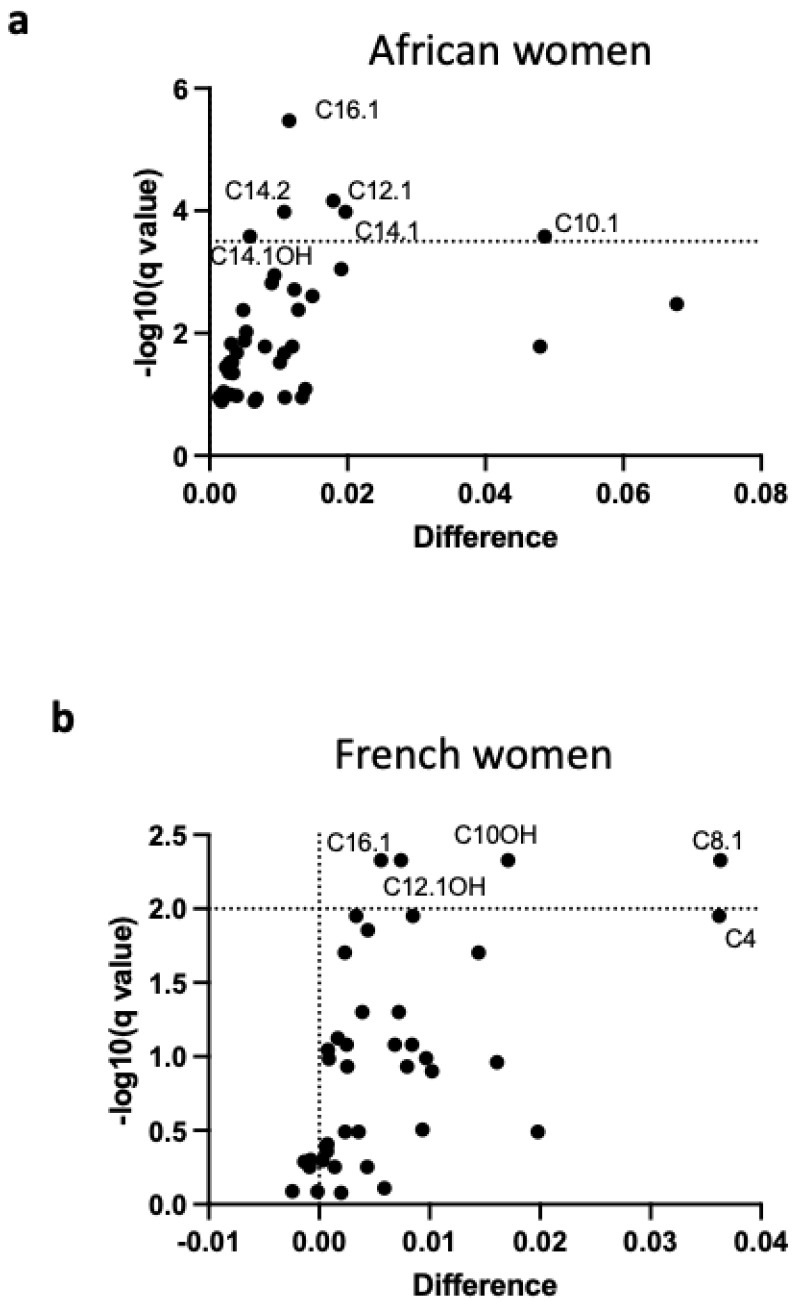
Volcano plots of increased metabolites of the short-, medium- and long-chain acylcarnitine (SC, MC, LC) categories associated with hypertension in the African (**a**) and French (**b**) populations. Dashed lines indicate limits of significance.

These correlations could not be investigated in French women as hypertension but not SBP was not reported. We investigated which acylcarnitine metabolites were specifically associated with higher SBP and/or reported hypertension in both populations. The most significant associations observed in African women were reported with MC and LC unsaturated acylcarnitines, including C10:2, C10:1, C12:1, C14:2, C14:1, C16:1 and C18:1, indicative of impaired beta-oxidation of mono-unsaturated MC and LC fatty acids (Figure S2 and Figure 4). The most significant associations were reported in MC and LC acylcarnitines, including C10 OH, C12, C12:1 OH, and C16.1, in the French women (Figure S3 and Figure 5). Acylcarnitines increased in both populations included C4, C8:1, C10:2, C12:1, C12, C12:1 OH, C12 OH, and C16:1, indicative of impaired beta-oxidation of mono-unsaturated MC and LC fatty acids as common prominent altered changes (Figure 6, Supplementary Table S2). The increased C12, C14:1, and C14 could reflect an impaired activity of the electron transfer flavoprotein (ETF) and electron transfer flavoprotein dehydrogenase (ETFDH); the increased C14 and C14.1 could reflect decreased activity of carnitine-acylcarnitine translocase and carnitine palmitoyl transferase II; the increased C14, C14:1, and C18:1 could reflect decreased activity of VLCAD and LCHAD/TFP; and the increase in C10:1 could reflect decreased activity of MCAD [24].

### 3.4. Predictors of Systolic Blood Pressure in Multivariate Analysis

We evaluated further the predictors of systolic blood pressure in multivariate analysis in the African population. We included in the model all the parameters that were significantly associated with SBP in the univariate analysis, including free carnitine, SC, MC, LC and specific metabolites of acylcarnitines. In this model, the remaining independent determinants were age, body mass index, glycemia, LC acylcarnitines, LDL-C and HDL-C (Table 3).

## 4. Discussion

Consistent with our working hypothesis, we found a significant difference in acylcarnitine markers of fatty acid oxidation according to components of MetS in both populations.

### 4.1. Association of Acylcarnitine Markers of Fatty Acid Oxidation with Obesity in African Women

Obesity was associated with short-, medium-, and long-chain acylcarnitines in African women. This association could not be assessed in the French population, which consisted solely of females with obesity. The concentrations of MC and LC acylcarnitines were increased in African women with MetS but not in French women, while SC acylcarnitines were increased in both populations. The selective increase in short-chain acylcarnitines according to MetS could be explained in part by the dietary profile of subjects, including the intake of food products of the Western diet adopted by African women. Baker et al. reported an alteration of β-oxidation in patients with obesity [20]. They measured the plasma and skeletal muscle metabolomic profiles before and after a 5-day high fat diet and found an accumulation of short- and medium-chain ACs in the 4 h postprandial condition [20]. A recent study conducted by Bouchard-Mercier et al. compared the metabolic profile in 210 healthy subjects according to two dietary patterns: the Prudent dietary pattern—characterized by higher intakes of vegetables, fruits, whole-grain products, and non-hydrogenated fats and lower intakes of refined grain products—and the Western dietary pattern—associated with higher intakes of refined grain products, desserts, sweets and processed meats [9]. In their study, the metabolic profile of the Western dietary pattern was associated with a specific metabolite signature characterized by increased levels of branched-chain AAs (BCAAs) and short-chain acylcarnitines. Another study comparing lean subjects versus subjects with obesity showed an elevation of C3, C5, C6, and C8:1 in subjects with obesity [7]. The contrasted dietary profile of both populations suggested that altered beta-oxidation may play a prominent role in the association of acylcarnitines with obesity in the African women. This hypothesis is consistent with the increased concentration of all acylcarnitine categories in humans with high caloric intake or a high-fat diet [25] and could reflect a combined role of the diet and impaired lipid oxidation in mitochondria [12]. In addition, experimental data showed that high-fat feeding of rodents leads to obesity and insulin resistance through impaired β-oxidation and accumulation of long- and medium-chain acylcarnitines and increased efflux of short-chain acylcarnitines from the mitochondria [4,26].

### 4.2. Potential Mechanisms Supported by Other Studies

The regulation of β-oxidation by the PGC1α/SIRT1 axis is among the potential mechanisms supported by other studies. The PGC1α/SIRT1 axis regulates mitochondrial biogenesis and expression of genes involved in the respiratory chain and β-oxidation. The decreased expression of the histone deacetylase SIRT1 is a prominent hallmark of obesity, which deactivates PGC1α through its hyperacetylation. It may also be produced by deficiencies in folate and vitamin B12, which are associated with DT2M risk and which are more prevalent in Africa than in Western countries [27]. C3 and C5 are the degradation products of branched-chain amino acids (BCAAs). Leucine and isoleucine can contribute at least 25% to the lipogenic acetyl-CoA pool, and valine and isoleucine can contribute up to 100% to the lipogenic propionyl-CoA pool [28]. Wolk et al. showed that propionyl carnitine concentrations reflect the propionyl-CoA and acetyl-CoA pools during adipogenesis [29]. In accordance with this observation, recent studies showed that odd-chain fatty acids can be produced by adipocytes and accumulate during cell differentiation. It is, in particular, the case of an excess of propionyl-CoA in patients with propionic acidemia or methylmalonic acidemia, which are diseases in relation to propionate catabolism [28,30]. Moreover, an increase in plasma C3 and C5 may reflect changes in the microbiome, as propionic and isobutyric acids can be produced by intestinal flora [31,32,33].

### 4.3. Association of Acylcarnitine’s Markers of Fatty Acid Oxidation with Insulin Resistance and T2DM

Several cohort studies have demonstrated that elevated circulating concentrations of BCAA may even serve as biomarkers for predicting the development of insulin resistance and T2DM [33,34,35,36,37]. Consistently, we observed an increase in SC acylcarnitines in diabetic French women, but we failed to find a relationship between SC acylcarnitines and diabetes in African women, in whom the prevalence of diabetes was much lower. These contrasted results may reflect differences in adaptive metabolic plasticity [28]. Dyslipidemia plays a role in the pathogenesis of insulin resistance [38]. We observed a high rate of high blood triglycerides in the subjects with MetS of both populations, but an association of triglycerides with SC acylcarnitines was only observed in French women. Few experimental and population studies have addressed the relationship between dyslipidemia and acylcarnitines. One study found an increase in medium-chain dicarboxylic acylcarnitines in mice fed a hyperlipidic diet [39]. The high flow circulating triglycerides lead to an increase in malonyl CoA levels in muscles and decreased oxidation of fatty acids in the liver [40]. The tissue accumulation of esterified fatty acids may contribute to an accumulation of metabolites derived from lipids, such as diacylglycerols, ceramides, ketones, prostaglandins, and other incompletely oxidized lipids, and contribute to altered insulin signaling [38,41,42].

### 4.4. Association of Acylcarnitine’s Markers of Fatty Acid Oxidation with Hypertension

Our most intriguing result was the elevation of SC, MC, and LC acylcarnitines in women with hypertension from both populations. The increased acylcarnitines reported in both populations, including C4, C8.1, C10.2, C12.1, C12, C12.1 OH, C12 OH, and C16.1, could reflect altered activities of the electron transfer flavoprotein (ETF) and electron transfer flavoprotein dehydrogenase (ETFDH), carnitine-acylcarnitine translocase and carnitine palmitoyl transferase II and activity of MCAD, VLCAD and LCHAD/TFP [24]. The underlying mechanisms should deserve further interest. Mels et al. showed a correlation between free L-carnitine and the systolic and diastolic pressures in Caucasian and African men [15]. A recent study in two African populations showed an increase in free carnitine and total carnitine in subjects with masked hypertension [43]. Several hypotheses could explain the link between acylcarnitines and SBP. The L-carnitine bio-availability could be decreased in subjects with hypertension whose increasing urbanized lifestyle results in changes in dietary patterns [44]. Increased SBP could increase the carnitine needs as a result of a higher demand for fatty acid catabolism to sustain cardiac function [45]. The association could also be related to a dysfunction of the NO pathway. A recent study showed that the eNO Knockout mouse ad low levels of carnitines, which reflect an impaired beta-oxidation of fatty acids [46]. Other studies suggest that mitochondrial dysfunction, secondary to a disruption of carnitine homeostasis, may also play a role in impaired NO signaling and the development of endothelial dysfunction associated with a variety of cardiovascular diseases [45]. However, these hypotheses are not consistent with our observation of an elevation of acylcarnitine metabolites related to impaired beta-oxidation of mono-unsaturated MC and LC fatty acids in both African and European populations. A recent study from Brittain et al. in patients with pulmonary arterial hypertension found a 1.5- to 2-fold increase in long-chain acylcarnitines palmitoyl carnitine (C16), stearoyl carnitine (C18), oleoyl carnitine (C18:1), and linoleoyl carnitine (C18:2) compared to controls [47]. Taken together, this and our studies suggest that human pulmonary or systemic hypertension is associated with specific abnormalities in fatty acid oxidations, which involve predominantly an impaired beta-oxidation of MC and LC fatty acids rather than a deficit in carnitine. Whether the impaired beta-oxidation of MC and LC fatty acids is a cause and/or consequence of high SBP should deserve further attention. Indeed, the increase in non-degraded unsaturated LC fatty acids by impaired beta-oxidation could lead to the increased synthesis of oxidized fatty acids involved in endothelial dysfunctions that contribute to hypertension [48].

### 4.5. Our Study Opened Perspectives and Suffered Some Limitations

We found an association between acylcarnitines and either systolic blood pressure or hypertension in two contrasted populations. Our data suggest, therefore, investigating further whether the association can be generalized in other populations despite distinct environments, lifestyles, dietary profiles and ethnicities.

We assumed that indexes of acylcarnitine concentrations were indicators of flux activities from pathways and enzymes of fatty acid oxidation according to previous data published in inherited disorders, but we did not validate their use by functional or genetic analyses. The lack of continuous BP data and the presence of treatment in the French cohort limit the strength and interpretability of the comparison between the two populations for this key outcome.

## 5. Conclusions

In conclusion, we observed an association of SC, MC, and LC acylcarnitines with MetS in the African population and of SC acylcarnitines with MetS in French women with morbid obesity and, more specifically, in the French population, an association of short-chain acylcarnitines with blood glucose and low HDL-cholesterol and high triglycerides. These associations could reflect impaired beta-oxidation by a cause–consequence cross-talk between dietary patterns and decreased expression of genes involved in beta-oxidation. We reported an association of increased SC, MC and LC acylcarnitines with systolic blood pressure in the two populations. The impaired beta-oxidation and related accumulation of mono-unsaturated fatty acids in women with high SBP could lead to the accumulation of oxidized fatty acids that contributes to endothelial dysfunction.

## Figures and Tables

**Table 1:** Clinical and biological characteristics of African and French women with or without metabolic syndrome.

		African Women			French Obese Women		
		Metabolic syndrome			Metabolic syndrome		
		No *n* = 343 (80.1%)	Yes *n* = 85 (19.9%)		No *n* = 86 (39.0%)	Yes *n* = 134 (61.0%)	
Components of metabolic syndrome, biological data and metabolic profiling		Mean ± SD		*p*	Mean ± SD		*p*
Clinical data, related or not to MetS							
Age, years		39 ± 1	49 ± 1	<0.0001	42 ± 1	47 ± 1	0.0031
Weight, kg	Yes	77.7 ± 0.9	90.5 ± 1.8	<0.0001	120.6 ± 1.9	121.5 ± 1.6	0.9385
Blood pressure (mmHg) /treated hypertension (%)	Yes	121 ± 18	150 ± 21	<0.0001	8 (9.4%)	77 (90.6%)	<0.0001
Hip circumference, cm	Yes	109.9 ± 0.7	118.1 ± 1.2	<0.0001	138.1 ± 1.9	137.5 ± 1.8	0.2164
Waist circumference, cm	Yes	89.8 ± 0.7	1013.1 ± 1.2	<0.0001	124.5 ± 2.0	124.8 ± 1.4	0.8877
Waist/hip ratio (WHR)	Yes	0.82 ± 0.0	0.88 ± 0.0	<0.0001	0.89 ± 0.02	0.93 ± 0.01	0.0068
BMI, kg/m^2^	Yes	30.4 ± 0.3	35.8 ± 0.7	<0.0001	45.3 ± 0.6	46.4 ± 0.6	0.4657
Fasting biochemistry							
Glucose, mmol/L	Yes	4.3 ± 0.0	4.6 ± 0.1	0.0609	4.8 ± 0.9	6.4 ± 0.2	<0.0001
Insulin, µU/mL	Yes	26.0 ± 0.8	27.2 ± 1.6	0.2690	35.2 ± 2.8	63.1 ± 5.1	<0.0001
HOMA-IR	Yes	5.1 ± 0.2	5.6 ± 0.4	0.0965	7.6 ± 0.6	19.2 ± 2.1	<0.0001
Total Cholesterol, g/L		1.7 ± 0.0	1.8 ± 0.1	0.0307	1.9 ± 0.0	1.8 ± 0.0	0.1613
HDL-Cholesterol, g/L	Yes	0.5 ± 0.0	0.4 ± 0.1	<0.0001	0.5 ± 0.0	0.4 ± 0.0	<0.0000
LDL-Cholesterol, g/L		1.1 ± 0.0	1.3 ± 0.0	<0.0001	1.2 ± 0.1	1.1 ± 0.0	0.0061
Triglycerides, g/L	Yes	0.6 ± 0.1	0.8 ± 0.0	<0.0001	1.1 ± 0.0	1.7 ± 0.1	<0.0001
ALAT, UI/L		12.1 ± 0.3	11.7 ± 0.6	0.7331	24.1 ± 2.1	43.4 ± 3.2	<0.0001
ASAT, UI/L		23.1 ± 0.5	22.9 ± 1.0	0.6639	22.9 ± 1.4	37.8 ± 2.4	<0.0001
Ratio ASAT/ALAT		2.2 ± 0.1	2.6 ± 0.3	0.6059	1.1 ± 0.1	1.0 ± 0.0	0.0721
Metabolic profiling							
Homocysteine, µmol/L		11.6 ± 0.3	12.6 ± 0.5	0.0009	13.5 ± 0.4	13.1 ± 0.4	0.2379
Folates, nmol/L		11.3 ± 2.1	6.2 ± 0.4	0.0022	10.6 ± 0.7	12.8 ± 0.8	0.0222
Vitamin B12, pmol/L		513.2 ± 13.3	458.2 ± 24.1	0.0055	229.5 ± 10.0	290.9 ± 35.3	0.0605
Total carnitine, µmol/L		41.5 ± 0.5	44.9 ± 11	0.0037	43.2 ± 1.2	47.5 ± 1.0	0.0037
Free carnitine, µmol/L		35.4 ± 0.5	38.3 ± 0.9	0.0116	33.0 ± 0.9	36.4 ± 0.9	0.0199
Short chain, µmol/L		0.85 ± 0.01	0.95± 0.02	0.0001	0.61 ± 0.01	0.73 ± 0.02	<0.0001
Medium chain, µmol/L		1.48 ± 0.04	1.83 ± 0.01	<0.0001	1.35 ± 0.03	1.51 ± 0.06	0.0907
Long chain, µmol/L		0.79 ± 0.01	0.88 ± 0.02	0.0004	0.80 ± 0.02	0.83 ± 0.02	0.3316
Very long chain, µmol/L		0.04 ± 0.00	0.04 ± 0.00	0.2523	0.02 ± 0.00	0.02 ± 0.00	0.6030

Baseline characteristics are presented according to absence (No) or presence (Yes) of metabolic syndrome as means ± SDs. Those used in the MetS stratification are indicated also by Yes. The Mann–Whitney U test was performed to assess differences (*p* values). ALAT: alanine amino transferase; ASAT: aspartate amino transferase; BMI: body mass index; HDL-C: high density lipoprotein; HOMA-IR: homeostasis model assessment for insulin resistance; LDL: low density lipoprotein; MetS: metabolic syndrome.

**Table 2:** Acylcarnitine’s concentrations and concentration indexes reflecting the fluxes of fatty acid oxidation in pathways from short-chain acyl-CoA dehydrogenase (SCAD), medium-chain acyl-CoA dehydrogenase (MCAD) and very-long-chain acyl-CoA dehydrogenase (VLCAD) activities in the African and French women with or without metabolic syndrome.

		African Women				French Women			
Acylcarnitines	Enzymes	No MetS	MetS	*p*	Adjusted *p*	No MetS	MetS	*p*	Adjusted *p*
C4, µmol/L	SCAD	0.09 ± 0.04	0.10 ± 0.05	**0.0032**	0.074	0.13 ± 0.04	0.17 ± 0.09	**0.0025**	**0.014**
C8, µmol/L	MCAD	0.18 ± 0.16	0.25 ± 0.26	**0.0001**	**0.015**	0.12 ± 0.16	0.12 ± 0.12	0.4476	0.095
C8/C10	MCAD	0.75 ± 0.25	0.70 ± 0.21	0.1734	0.059	0.71 ± 0.13	0.73 ± 0.13	0.3322	0.872
C14:1, µmol/L	VLCAD	0.08 ± 0.04	0.10 ± 0.05	**0.0001**	**0.004**	0.07 ± 0.03	0.07 ± 0.03	0.5442	0.333
C0/(C16:1 + C18)	CPT1	202.64 ± 3.88	203.66 ± 6.86	0.6991	0.962	197.83 ± 5.23	196.84 ± 5.77	0.8485	0.231
C0/C2	Even-numbered oxidation	4.86 ± 1.52	4.78 ± 1.54	0.2022	0.405	4.95 ± 1.51	4.81 ± 1.61	0.4157	0.984
(C2 + C3)/C0	Overall activity ß-oxidation	0.21 ± 0.02	0.23 ± 0.02	0.2775	**0.033**	0.22 ± 0.06	0.24 ± 0.08	0.3745	0.829
(C3 + C5)/ Total C	BCAA metabolism	0.005 ± 0.001	0.005 ± 0.002	0.1430	0.055	0.008 ± 0.002	0.008 ± 0.002	0.2346	0.127

Acylcarnitines are presented according to absence (No) or presence (Yes) of metabolic syndrome as means ± SDs. The Mann–Whitney U test was performed to assess differences (*p*-values). BCAA: branched amino acids; CPT1: carnitine palmitoyl transferase 1; C0: free carnitine; C4: isobutyryl acylcarnitine; C8: octanoyl acylcarnitine; C10: decanoyl acylcarnitine; C14:1: tetradecenoyl acylcarnitine; C16:1: hexadecanoyl acylcarnitine; C18: octadecanoyl –(stearyl-) acylcarnitine; MCAD: medium-chain acyl-CoA dehydrogenase; SCAD: short-chain acyl-CoA dehydrogenase; VLCAD: very-long-chain acyl-CoA dehydrogenase. Significant *p*-values are indicated in bold.

**Table 3:** Multivariate logistic regression analysis of determinants of high systolic blood pressure in Dantokpa cohort.

	African Women		
	Odds ratio (95% C.I.)	z	*p*
Age	1.07 (1.06–1.11)	7.46	0.000
Glucose	18.12 (2.41–135.71)	2.82	0.005
LDL-C	2.36 (1.21–4.60)	2.36	0.011
LC-AC	5.42 (1.60–18.43)	2.71	0.007

LDL-C: low density lipoprotein cholesterol; HDL-C: high density lipoprotein cholesterol; LC-AC, long-chain acylcarnitines. The model included all the parameters that were significantly associated with SBP in the univariate analysis, including free carnitine and all categories of acylcarnitines highlighted in Figures S2 and S3.

## Data Availability

No new data were created or analyzed in this study.

## Supplementary Materials

The following supporting information can be downloaded at: https://www.mdpi.com/article/10.3390/nu18071137/s1. Table S1: Blood concentrations of acylcarnitines in African women of Dantokpa cohort and French obese women of the ALDEPI/OBESEPI cohort according to metabolic syndrome. Table S2: Blood concentrations of acylcarnitines in African women of Dantokpa cohort and French obese women of the ALDEPI/OBESEPI cohort according to high blood pressure or hypertension. Figure S1. Circos plots of acylcarnitines concentrations according to presence or absence of metabolic syndrome (MetS) in African and French populations. Figure S2. Metabolites of the Short, medium and long chain acylcarnitines (SC, MC, LC) categories according to hypertension (blood pressure, BP) in the African population. Figure S3. Metabolites of the Short, medium and long chain acylcarnitines (SC, MC, LC) categories according to hypertension (blood pressure, BP) in the French population.

## Abbreviations

BCAAs, branched-chain amino acids; CVD, cardiovascular disease; IDF, International Diabetes Federation; MetS, metabolic syndrome; SC, short chain; MC, medium chain; MetS, metabolic Ssyndrome; LC, long chain; DT2M, type 2 diabetes mellitus; WHR, waist-to-hip ratio.
