# Supplementary material for: Acylcarnitines Are Associated with Metabolic Syndrome and Hypertension in Two Contrasted Obese Populations
Source: Nutrients. 2026 Apr 1;18(7):1137. doi: 10.3390/nu18071137 (PMC13075005; doi:10.3390/nu18071137)

Table S1: Blood concentrations of acylcarnitines in African women of Dantokpa cohort and French obese women of the ALDEPI/OBESEPI cohort according to metabolic syndrome

Acylcarnitines	African women			French obese women		
	Metabolic syndrome			Metabolic syndrome		
	No<br>*n*=343<br>(80.1%)	Yes<br>*n*=85<br>(19.9%)		No<br>*n*=77<br>(35.0%)	Yes<br>*n*=143<br>(65.0%)	
	Mean ± SD		*P*	Mean ± SD		*P*
C3, µmol/L	0.74 ± 0.07	0.20 ± 0.09	0.0016	0.27 ± 0.09	0.32 ± 0.13	0.0072
C4, µmol/L	0.08 ± 0.04	0.10 ± 0.05	0.0032	0.13 ± 0.06	0.16 ± 0.09	0.0014
C5, µmol/L	0.09 ± 0.04	0.10 ± 0.05	0.0023	0.09 ± 0.02	0.11 ± 0.04	<0.0001
C5.1, µmol/L	0.02 ± 0.01	0.03 ± 0.01	0.0049	0.01 ± 0.00	0.01 ± 0.00	0.2916
C6, µmol/L	0.07 ± 0.04	0.10 ± 0.05	<0.0001	0.08 ± 0.03	0.10 ± 0.06	0.0046
C6dc, µmol/L	0.03 ± 0.01	0.04 ± 0.01	0.0721	0.17 ± 0.04	0.17 ± 0.05	0.6046
C8, µmol/L	0.17 ± 0.16	0.25 ± 0.26	0.0001	0.10 ± 0.04	0.13 ± 0.17	0.2479
C8.1, µmol/L	0.07 ±0.03	0.09 ± 0.04	0.0001	0.13 ± 0.06	0.15 ± 0.07	0.0035
C10, µmol/L	0.24 ± 0.19	0.34 ± 0.26	<0.0001	0.14 ± 0.06	0.18 ± 0.19	0.3593
C10.1, µmol/L	0.16 ± 0.10	0.22 ± 0.15	<0.0001	0.08 ± 0.02	0.10 ± 0.06	0.0601
C12, µmol/L	0.09 ± 0.04	0.12 ± 0.06	<0.0001	0.06 ± 0.02	0.07 ± 0.03	0.0110
C12.1oh, µmol/L	0.08 ± 0.04	0.09 ± 0.05	0.0903	0.02 ± 0.00	0.02 ± 0.01	0.0972
C14, µmol/L	0.04 ± 0.01	0.05 ± 0.02	0.0397	0.03 ± 0.01	0.04 ± 0.01	0.0290
C16, µmol/L	0.14 ± 0.04	0.15 ± 0.04	0.1724	0.13 ± 0.04	0.14 ± 0.04	0.1585
C18	0.04 ± 0.01	0.05 ± 0.01	0.1067	0.04 ± 0.01	0.04 ± 0.01	0.2429
C18.1, µmol/L	0.09 ± 0.03	0.10 ± 0.04	0.2255	0.18 ± 0.05	0.18 ± 0.06	0.6987
C18.2oh, µmol/L	0.04 ± 0.01	0.05 ± 0.02	0.0031	0.03 ± 0.01	0.03 ± 0.01	0.2451

Blood concentrations of Acylcarnitines are presented according to absence (No) or presence (Yes) of metabolic syndrome as means ± SDs. The Mann-Whitney U test was performed to assess differences (*P* values).

Table S2: Blood concentrations of acylcarnitines in African women of Dantokpa cohort and French obese women of the ALDEPI/OBESEPI cohort according to high blood pressure or hypertension

Acylcarnitines	African women			French obese women		
	Hypertension			Hypertension		
	No	Yes		No	Yes	
	n=343 (80.1%)	*n*=85 (19.9%)		*n*=77 (35.0%)	*n*=143 (65.0%)	
	Mean ± SD		*P*	Mean ± SD		*P*
C3, µmol/L	0.17 ± 0.07	0.19 ± 0.07	0.1108	0.29 ± 0.12	0.31 ± 0.12	0.2162
C4, µmol/L	0.08 ± 0.04	0.09 ± 0.05	0.0166	0.14 ± 0.07	0.18 ± 0.09	<0.0001
C5, µmol/L	0.09 ± 0.05	1.00 ± 0.05	0.0684	0.10 ± 0.3	0.12 ± 0.03	0.0025
C5.1, µmol/L	0.02 ± 0.01	0.02 ± 0.01	0.0132	0.01 ± 0.00	0.01 ± 0.00	0.4057
C4OH, µmol/L	0.03 ± 0.01	0.04 ± 0.01	0.0146	0.05 ± 0.03	0.06 ± 0.03	0.0015
C6	0.07 ± 0.04	0.09 ± 0.05	0.0028	0.08 ± 0.05	0.09 ± 0.04	0.1302
C5OH	0.05 ± 0.02	0.05 ± 0.02	0.0345	0.03 ± 0.01	0.03 ± 0.01	0.8842
C6dc, µmol/L	0.03 ± 0.01	0.03 ± 0.01	0.1916	0.03 ± 0.01	0.04 ± 0.02	0.0001
C8, µmol/L	0.17 ± 0.17	0.22 ± 0.21	<0.0001	0.12 ± 0.17	0.12 ± 0.07	0.0444
C8.1, µmol/L	0.10 ± 0.03	0.11 ± 0.03	0.0077	0.13 ± 0.06	0.17 ± 0.08	0.0002
C10, µmol/L	0.24 ± 0.20	0.31 ± 0.22	<0.0001	0.17 ± 0.19	0.17 ± 0.09	0.0851
C10.1, µmol/L	0.16 ± 0.10	0.21 ± 0.14	0.0820	0.09 ± 0.05	0.10 ± 0.04	0.0221
C12, µmol/L	0.09 ± 0.04	0.11 ± 0.05	<0.0001	0.06 ± 0.02	0.07 ± 0.02	0.0081
C12.1oh, µmol/L	0.02 ± 0.01	0.03 ± 0.01	0.0001	0.02 ± 0.00	0.03 ± 0.01	0.0235
C14, µmol/L	0.04 ± 0.01	0.04 ± 0.01	0.0540	0.03 ± 0.00	0.04 ± 0.01	0.0128
C16, µmol/L	0.14 ± 0.04	0.15 ± 0.04	0.0225	0.13 ± 0.04	0.14 ± 0.04	0.0256
C18, µmol/L	0.03 ± 0.01	0.04 ± 0.01	0.0110	0.04 ± 0.01	0.04 ± 0.01	0.3182
C18.1, µmol/L	0.09 ± 0.03	0.11 ± 0.03	0.0032	0.18 ± 0.05	0.19 ± 0.06	0.0852
C18.2oh, µmol/L	0.04 ± 0.01	0.05 ± 0.02	0.0119	0.03 ± 0.01	0.03 ± 0.01	0.3237

Blood concentrations of Acylcarnitines are presented according to absence (No) or presence (Yes) of metabolic syndrome as means ± SDs. The Mann-Whitney U test was performed to assess differences (*P* values).

Figure S1. Circos plots of acylcarnitines concentrations according to presence or absence of metabolic syndrome (MetS) in African and French populations. Circos plots represent pairwise correlations between acylcarnitines in the whole population and according to metabolic syndrome status (No MetS and MetS). Each segment corresponds to an individual acylcarnitine, and links indicate significant correlations, with thickness proportional to correlation strength. The MetS group shows a reorganization of the correlation network compared to the No MetS group

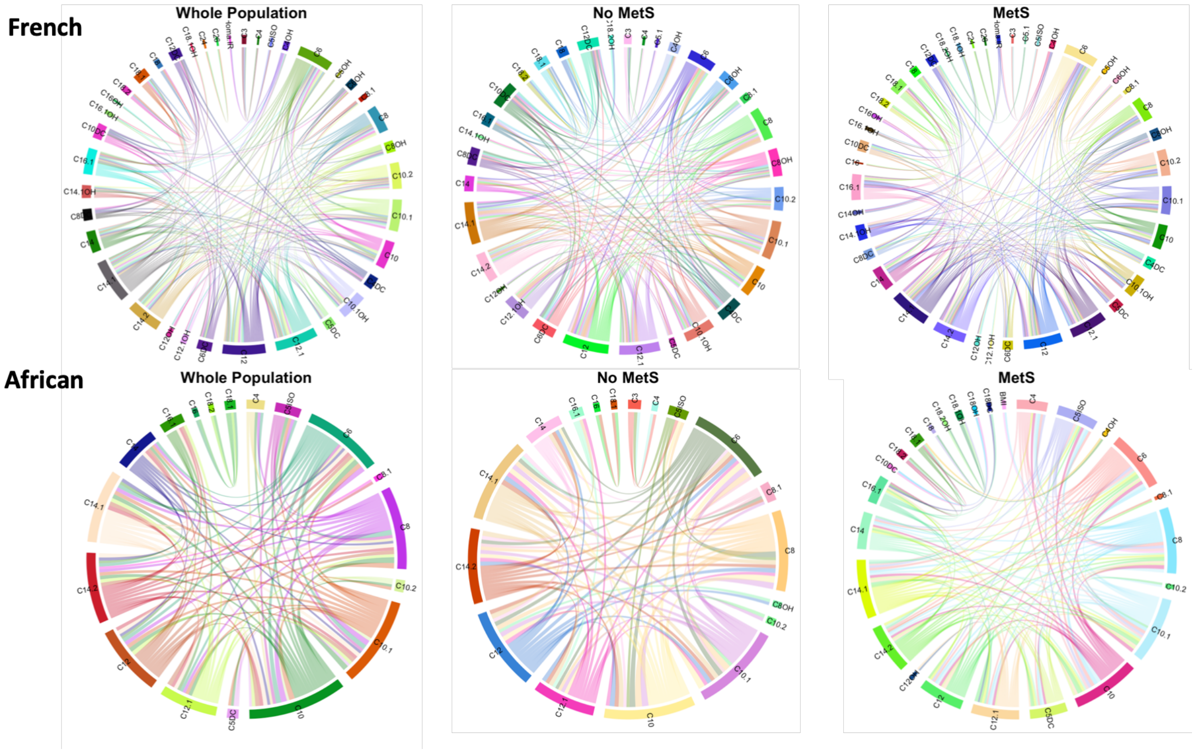

Figure S2. Metabolites of the Short, medium and long chain acylcarnitines (SC, MC, LC) categories according to hypertension (blood pressure, BP) in the African population. Differences were assessed by Mann-Whitney U test. Statistical significances *$p < 0.05$, **$p < 0.01$, ***$p < 0.001$, ****$p < 0.0001$.

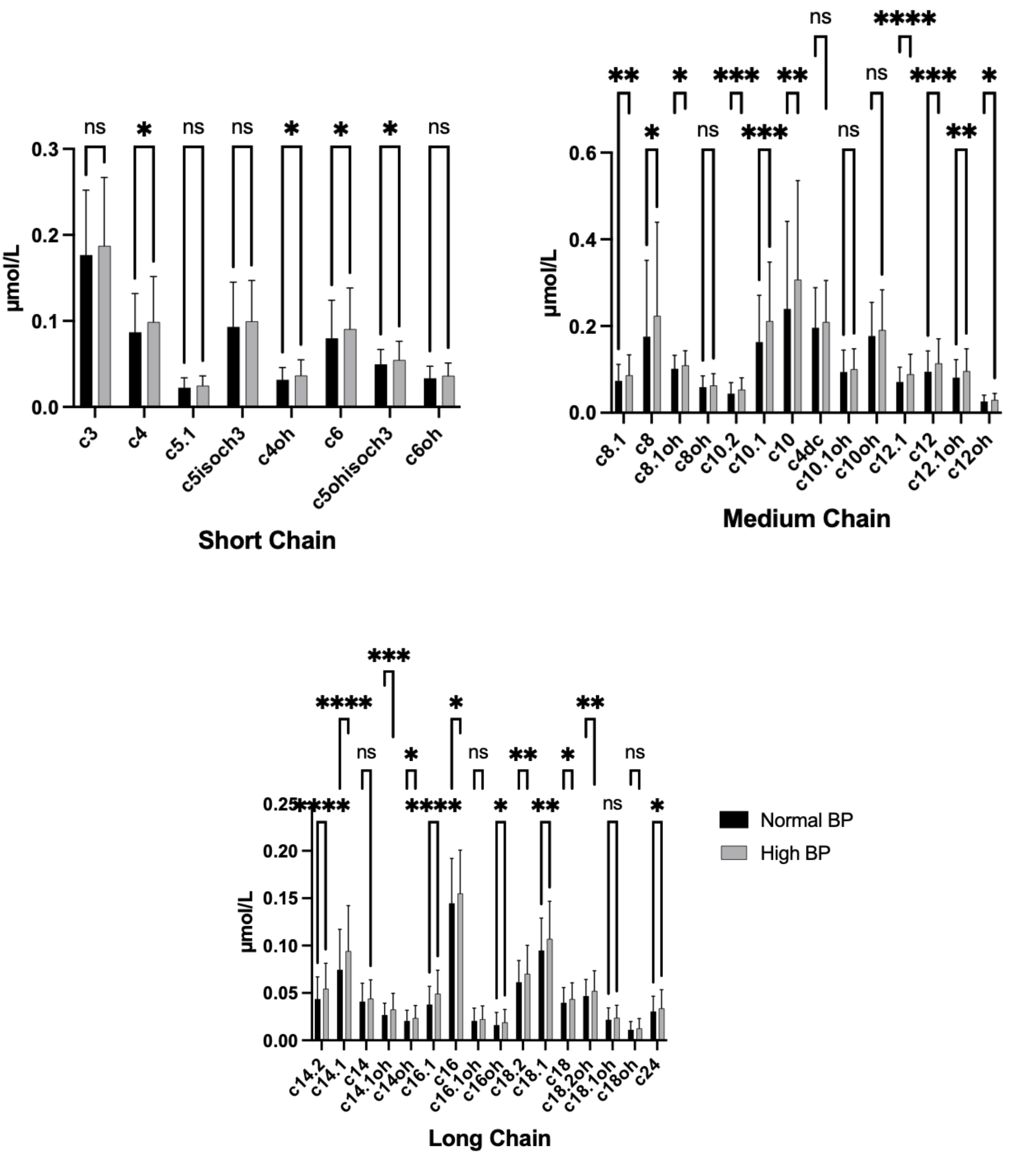

Figure S3. Metabolites of the Short, medium and long chain acylcarnitines (SC, MC, LC) categories according to hypertension (blood pressure, BP) in the French population. Differences were assessed by Mann-Whitney U test. Statistical significances $*p < 0.05$, $**p < 0.01$, $***p < 0.001$, $****p < 0.0001$.

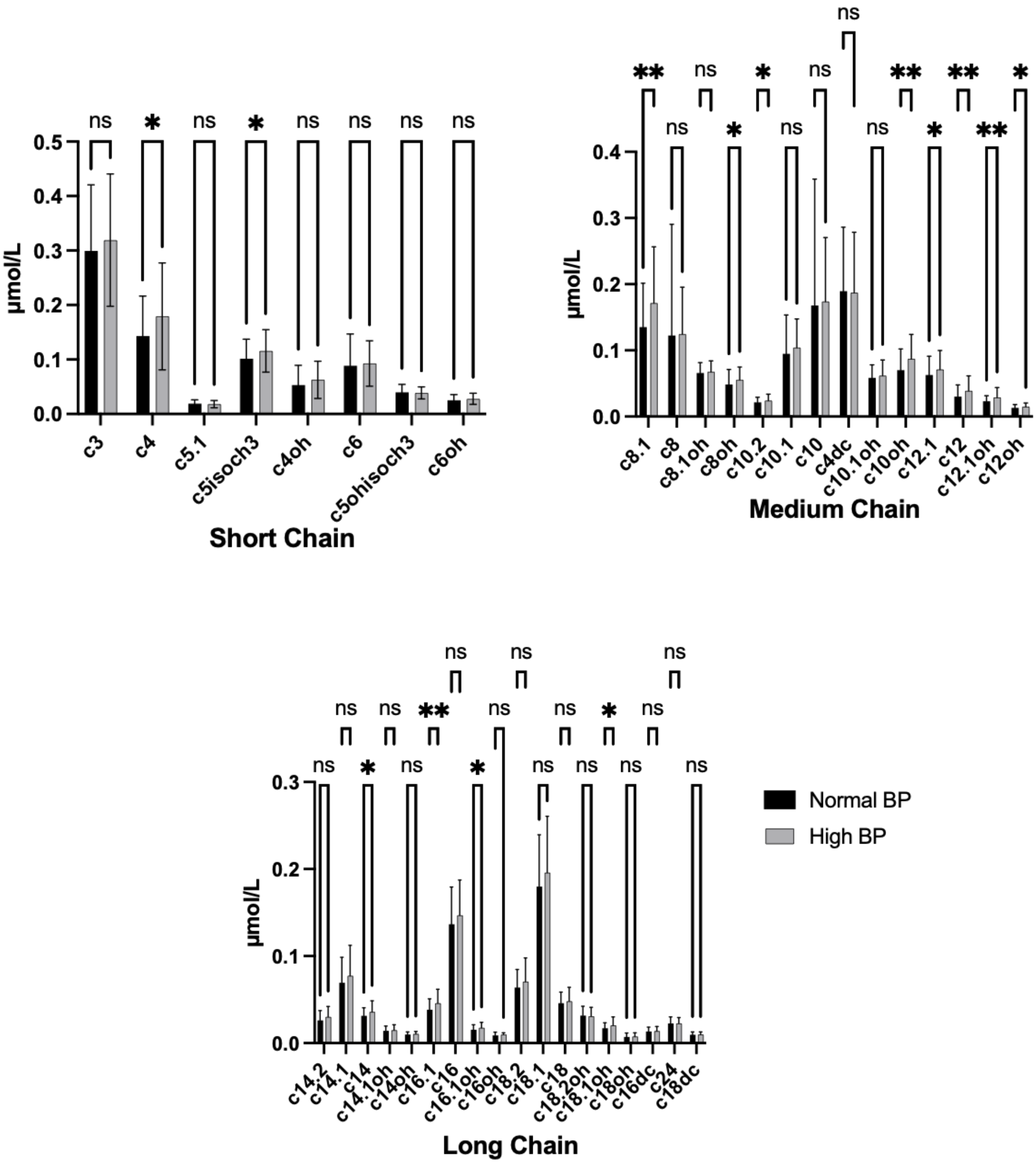

Supplement: Supplementary file 1 [file nutrients-18-01137-s001.zip › nutrients-4077997-supplementary.pdf]
